# Loss of Metabolic Flexibility in the Failing Heart

**DOI:** 10.3389/fcvm.2018.00068

**Published:** 2018-06-06

**Authors:** Qutuba G. Karwi, Golam M. Uddin, Kim L. Ho, Gary D. Lopaschuk

**Affiliations:** Cardiovascular Research Centre, University of Alberta, Edmonton, AB, Canada

**Keywords:** fatty acid oxidation, glucose oxidation, ketone oxidation, cardiac metabolism, heart failure, insulin resistant

## Abstract

To maintain its high energy demand the heart is equipped with a highly complex and efficient enzymatic machinery that orchestrates ATP production using multiple energy substrates, namely fatty acids, carbohydrates (glucose and lactate), ketones and amino acids. The contribution of these individual substrates to ATP production can dramatically change, depending on such variables as substrate availability, hormonal status and energy demand. This “metabolic flexibility” is a remarkable virtue of the heart, which allows utilization of different energy substrates at different rates to maintain contractile function. In heart failure, cardiac function is reduced, which is accompanied by discernible energy metabolism perturbations and impaired metabolic flexibility. While it is generally agreed that overall mitochondrial ATP production is impaired in the failing heart, there is less consensus as to what actual switches in energy substrate preference occur. The failing heart shift toward a greater reliance on glycolysis and ketone body oxidation as a source of energy, with a decrease in the contribution of glucose oxidation to mitochondrial oxidative metabolism. The heart also becomes insulin resistant. However, there is less consensus as to what happens to fatty acid oxidation in heart failure. While it is generally believed that fatty acid oxidation decreases, a number of clinical and experimental studies suggest that fatty acid oxidation is either not changed or is increased in heart failure. Of importance, is that any metabolic shift that does occur has the potential to aggravate cardiac dysfunction and the progression of the heart failure. An increasing body of evidence shows that increasing cardiac ATP production and/or modulating cardiac energy substrate preference positively correlates with heart function and can lead to better outcomes. This includes increasing glucose and ketone oxidation and decreasing fatty acid oxidation. In this review we present the physiology of the energy metabolism pathways in the heart and the changes that occur in these pathways in heart failure. We also look at the interventions which are aimed at manipulating the myocardial metabolic pathways toward more efficient substrate utilization which will eventually improve cardiac performance.

## Introduction

Heart failure is a major cause of death and disability, with more than 26 million people diagnosed with heart failure worldwide (1). The mortality rate of heart failure is approximately 30% within 5 years following diagnosis (2). Thus, heart failure presents a tremendous burden on society, the health care system and the economy (3, 4). While pharmacological management, primary prevention and earlier diagnosis have dramatically improved in the last 20 years, there is still a high morbidity and mortality associated with heart failure.

Different energy substrates, namely fatty acids, carbohydrates (glucose and lactate), ketones and amino acid, contribute differently to meet the high energy demand of the heart. Fatty acid oxidation is the biggest contributor to ATP production (~40–60%) while carbohydrates metabolism generates the remainder (~20–40%). The heart has a “metabolic flexibility,” a virtue which allows it to switch between these energy substrates according to the workload, availability of the substrates, and the hormonal status. There is a general consensus that this metabolic flexibility is impaired in heart failure which affects ATP production and, consequently, cardiac contractility. Another important dimension of the metabolic flexibility is the effect of inotropic agents on energy substrates mobilization and use which occurs in response to an increase in heart work. It has been demonstrated that adrenergic stimulation, using epinephrine, enhances glycogenolysis and increases glucose oxidation in the normal rat heart (5, 6). Nevertheless, whether this form of metabolic flexibility is also impaired in the failing heart has yet to be characterized. As a disease, the complexity of heart failure is represented by differences in etiology, severity and the presence of comorbidities, such as hypertension, diabetes and obesity. Despite its diverse nature, the failing heart is characterized by its energy deficient state, as evidenced by a 30–40% reduction in ATP production and reduced phosphocreatine/ATP (PCr/ATP) ratio (7–10). This energy-starved state may be due to mitochondrial dysfunction as a result of reactive oxygen species generation, compromised bioenergetics and impaired mitochondrial fission and fusion [see De Jong et al. (11) and Neubauer (10) for reviews]. Furthermore, mitochondrial dysfunction also directly affects myocardial energy substrate metabolism further aggravating the energy crisis. Alterations in myocardial energy substrate metabolism are discernible in the failing heart as a result of compromised mitochondrial oxidative phosphorylation [see Lopaschuk et al. (12) for review]. This results in an increased reliance on glycolysis and decreased glucose oxidation to produce the required energy (13, 14). While ketone oxidation increases in the failing heart (15–17), fatty acid oxidation has been suggested to decrease (10, 18–20), or not change (13, 21). Regardless, these metabolic shifts in heart failure can negatively affect cardiac contractility and myocardial energy management, leading to decreased metabolic flexibility at a time when the heart needs it most. While the pathological consequences of changes to fatty acid and glucose metabolism have been extensively studied, the alterations and implications of altered ketone body metabolism during heart failure remain unclear even though the heart uses the most ketone bodies per unit mass (22, 23). Lastly, there is an increasing amount of evidence suggesting that restoring metabolic flexibility by enhancing glucose oxidation directly (24) or indirectly through inhibition of fatty acid oxidation (25) could improve myocardial ATP production, improve cardiac function and limit cardiac remodeling (12, 13, 26, 27).

This review will focus on the alterations in fatty acid oxidation, carbohydrate metabolism and ketone metabolism that occur in the heart in the setting of ischemic heart failure. Amino acid metabolism is out of the scope of this review and the reader is referred to other reviews about the subject (28, 29). In addition, we will illustrate how energy metabolism in ischemic heart failure is controlled by the interplay of transcriptional regulation, post-translational modifications, and cytosolic/mitochondrial signaling kinases. We will also critically appraise some of the published data and discuss the controversies surrounding energy substrate preference in the failing heart. Lastly, we will briefly discuss the advances in pharmacological interventions which target myocardial energy metabolism as an approach to treat heart failure.

## Cardiac metabolism in the healthy heart

**Figure 1:**
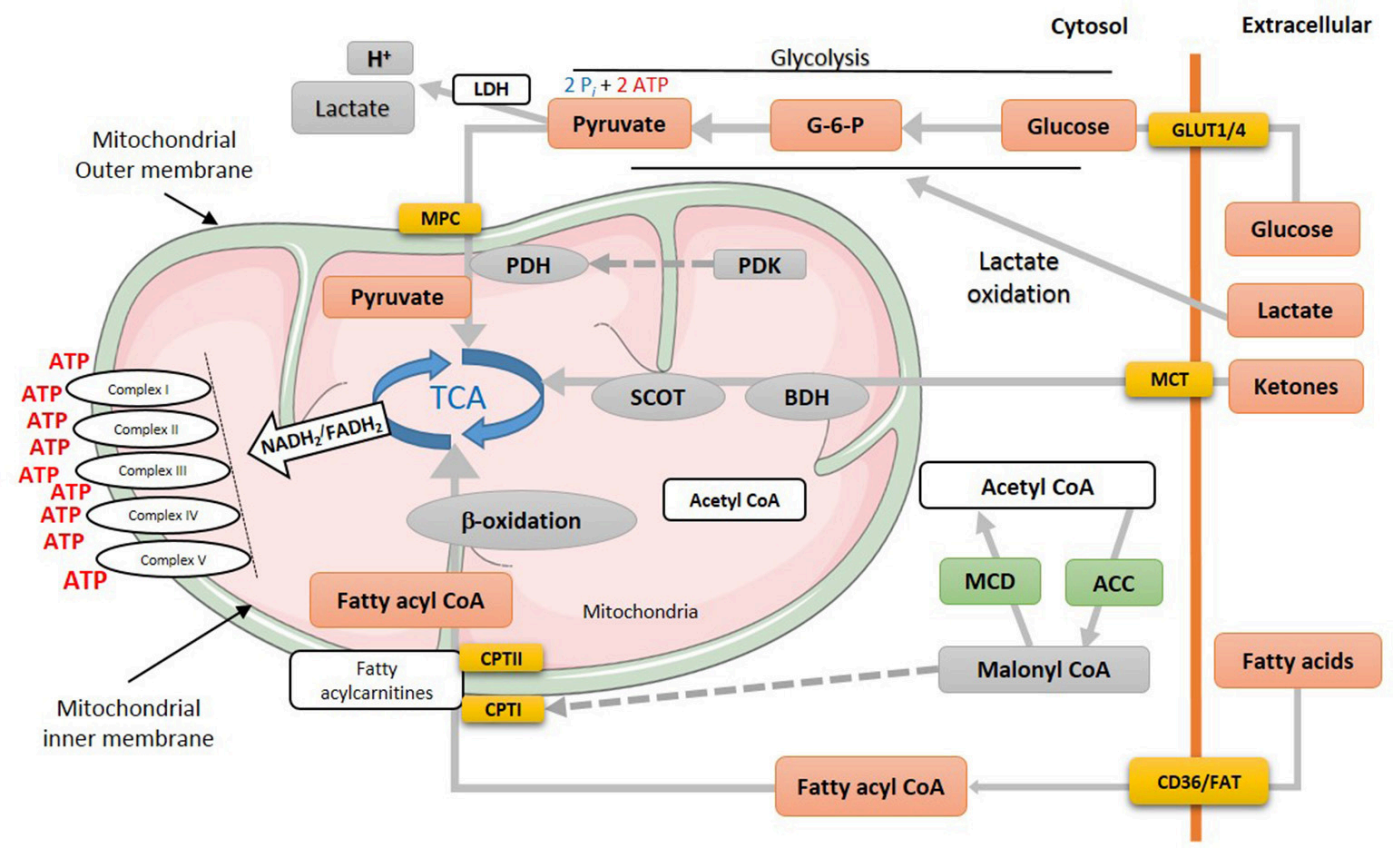
Energy metabolism in normal heart. Various metabolic pathways contribute to mitochondrial ATP production in the heart. Mitochondrial ATP production uses mostly fatty acids, glucose and ketones as a fuel source. The production of acetyl CoA by fatty acid ß-oxidation first requires the mitochondrial uptake of fatty acids via a carnitine carrier system. Oxidation of glucose involves the production of pyruvate via glycolysis, which produces acetyl CoA for the TCA cycle via PDH. Acetyl CoA production by ketone body oxidation is facilitated by BDH and SCOT. MPC, mitochondrial pyruvate career; PDH, pyruvate dehydrogenase; PDK, pyruvate dehydrogenase kinase; TCA, tricarboxylic acid cycle; SCOT, succinyl-CoA-3-oxaloacid CoA transferase; BDH, β-hydroxybutyrate dehydrogenase; CPT, Carnitine palmitoyltransferase; MCD, malonyl CoA dehydrogenase; ACC, acetyl CoA carboxylase; MCT, monocarboxylate transporter; CD36, cluster of differentiation; FAT, fatty acid translocase; GLUT, glucose transporter type (1 or 4).

Cardiomyocytes have a virtue of metabolic adaptability as they can omnivorously utilize different energy substrates, including fatty acids, carbohydrates (glucose and lactate), ketones and amino acids, to meet the high energy demand of the heart and to sustain contractile function. Due to this metabolic flexibility, the heart's substrate preference can rapidly change based on the availability of energy substrates supplied to the heart, hormonal status and the changing workload of the heart. Mitochondrial oxidative phosphorylation produces the majority of the high-energy phosphates needed to sustain contractile function (Figure 1). Of this, fatty acid oxidation is the biggest contributor to mitochondrial oxidative metabolism, providing approximately 40–60% of the total energy produced, with the oxidation of glucose, lactate, ketone and amino acids providing the remaining 20–40%. To better understand the use of each substrate, first, we will discuss glucose, fatty acid, and ketone metabolism in the normal healthy heart.

## Glucose metabolism

Locke and Rosenheim in 1907 were the first to study myocardial glucose uptake in an isolated rabbit heart model (30). Thirty years later, Albert Szent-Gyorgyi, Hans A. Krebs and William A. Johnson reported the conversion of pyruvate to succinate, unearthing the Krebs cycle (31). The uptake of glucose into the cardiomyocyte occurs though insulin-independent (GLUT1) and insulin-dependent (GLUT4) transporters. Transported glucose is first phosphorylated by hexokinase to glucose-6-phosphate (G-6-P), which can then undergo different metabolic pathways. G-6-P can be used as a substrate for glycolysis to produce pyruvate, NADH and 2 ATP (Figure 1) or it can enter the hexosamine biosynthesis pathway. In addition, G-6-P can be used for glycogen synthesis or shuttled to the pentose phosphate pathway (32). Glycolysis-derived pyruvate can either be converted to lactate by lactate dehydrogenase (LDH) or alternatively transferred to the mitochondria matrix by the mitochondrial pyruvate carrier (MPC) (33) (Figure 1). In the mitochondrial matrix, pyruvate dehydrogenase (PDH), the rate limiting enzyme of glucose oxidation, catalyzes the conversion of pyruvate to acetyl CoA which feeds into the tricarboxylic acid (TCA) cycle. Mitochondrial pyruvate can also supply the TCA cycle with essential intermediates, namely oxaloacetate and malate, via carboxylation. The enzymatic activity of PDH can be inhibited through phosphorylation by PDH kinase (PDK) and be reactivated by PDH phosphatase (34).

In terms of energy producing efficiency, oxidation of each glucose molecule requires 6 O_2_ molecules and produces 31 high-energy phosphate (ATP) molecules. Accordingly, glucose's phosphate/oxygen (P/O) ratio is 2.58, making glucose the most efficient energy substrate. Glucose metabolism also produces two high energy phosphate (P_*i*_) molecules by converting glucose to pyruvate through glycolysis (Figure 1).

## Fatty acid oxidation

Beta-oxidation of fatty acids was first proposed by a work of Knoop (35) which was later verified by a following study by Dakin (36). After a half century, Richard Bing and colleagues discovered that the human heart prefers fatty acids for respiration (37). Later in 1963, Philip Randle and his group (38) proposed that glucose and fatty acids are working in a concert to meeting the high energy demand of the heart, which is later known as the Randle cycle. The source of fatty acids for mitochondrial oxidative metabolism originates from circulating free fatty acids (FFAs) bound to albumin and/or released from triacylglycerols (TAG) contained in chylomicrons or very-low-density lipoproteins (VLDL) (39–41). Some fatty acids also originate from intracellular TAG stores (42). Extracellular fatty acids are transported to the cardiomyocytes through passive diffusion or via the fatty acid transport protein (FATP) or fatty acid translocase (FAT, CD36) (39, 43, 44). Once FFAs are in the cytosol, they are esterified to long-chain fatty acyl CoA via an ATP-dependent pathway initiated by a family of fatty acyl-CoA synthase enzymes (45). The majority (90%) of long-chain fatty acyl CoAs are shuttled directly to the mitochondria, facilitated by carnitine palmitoyltransferase isomers (CPTI and CPTII), with the remainder stored in the myocardial TAG reservoir. CPTI converts long chain fatty acyl CoAs to long chain acylcarnitine in the outer mitochondrial membrane which can then be converted back to long chain fatty acyl CoA by CPTII in the inner mitochondrial membrane (27, 46–48). Flux of fatty acids through CPT1 is regulated by malonyl CoA, a potent inhibitor of CPT1 (49–51). Malonyl CoA levels in turn are primarily dependent on a balance between acetyl CoA synthesis by acetyl CoA carboxylase (ACC) (52–54) and degradation by malonyl CoA decarboxylase (MCD) (27, 55, 56) (Figure 1). It is worth mentioning that via a carnitine shuttle system, mitochondrial acetyl CoA can be shuttled back to the cytosol to further affect malonyl CoA levels (57). Additionally, citrates produced from the TCA cycle can also translocate to the cytosolic compartment where it can activate ACC1, produce more malonyl CoA, and send a feedback response to decrease fatty acid uptake into the mitochondria (27).

In terms of ATP production efficiency, the oxidation of a representative fatty acid molecule, namely palmitate, consumes 23 molecules of O_2_ and produces 105 high-energy phosphate (ATP) molecules. This means that fatty acids are the least efficient energy substrate with a P/O ratio of 2.33 compared to glucose oxidation (2.58). Moreover, esterification of fatty acid to fatty acyl CoA requires 2 ATP-derived P_i_. Similarly, cytosolic processing of fatty acid to fatty acyl CoA or TAG as well as reconverting TAG to fatty acid also consumes 2 P_i_. This further reduces the total energy profit from fatty acid oxidation.

## Ketone body oxidation

Ketone bodies are produced during times of fasting and starvation via hepatic ketogenesis, producing three types of ketone bodies: acetone, β-hydroxybutyrate (βOHB) and acetoacetate. Acetone, present in low abundance, is excreted by exhalation while βOHB and acetoacetate are the main ketone bodies circulating in our blood (58, 59). The predominant amount of ketogenesis occurs in the liver and the heart itself cannot produce ketones. Therefore, once circulating ketones reach the heart, ketones can enter the cardiomyocytes and be transported to the mitochondrial matrix where βOHB is oxidized into acetoacetate by βOHB dehydrogenase (BDH1), after which acetoacetate is activated by succinyl-CoA to acetoacetyl-CoA by the rate limiting enzyme, succinyl-CoA:3-oxoacid-CoA-transferase (SCOT) which is encoded by the gene *Oxct1*. Lastly, acetyl-CoA acetyltransferase converts acetoacetyl-CoA into acetyl-CoA (60, 61). Acetyl-CoA can then enter the tricarboxylic acid cycle and electron transport chain to generate ATP. Regulation of ketone oxidation enzymes in the heart remains poorly understood although hepatic BDH1 has been shown to be post-translationally modified by SIRT5 (62) and cardiac SCOT may be regulated by residue-specific nitration in the settings of diabetes (63, 64) and aging (65).

Ketone body as a fuel for the heart has long been recognized (37, 66). Barnes and Waters along with their associates (67, 68) first demonstrated that the heart can use ßOHB in 1938. In 1965, Rudolph et al. (69) showed that ßOHB and acetoacetate accounted for 2.6% ± 0.3% and 6.0% ± 1.0%, respectively, of the heart's total oxidative metabolism. Despite minor contribution to the total ATP production compared to fatty acids or glucose (12), myocardial oxidation of ketones can increase significantly in response to changes in the arterial concentration (69). Therefore, blood ketone levels are a key factor in determining myocardial ketone body oxidation rates and influencing the heart's metabolic flexibility. Lastly, it is important to consider, since the P/O ratio of ßOHB, is 2.50, this would make ketones less efficient than glucose but more efficient than palmitate.

## Cardiac metabolism in the failing heart

In heart failure, cardiac energy metabolism is compromised due to impaired cardiac ATP production, metabolic flexibility, mitochondrial TCA cycle activity and overall oxidative metabolism (9, 70, 71). More specifically, impaired mitochondrial function and oxidative capacity (10, 19, 72) results in reduced ATP production by up to 40% compared to the normal heart. In humans, the phosphocreatine/ATP ratio has been shown to decrease in heart failure patients (73–75), alongside impaired mitochondrial electron transport chain activity (19, 76). However, the exact energy metabolic profile of heart failure remains controversial as there is still discrepancy regarding substrate preferences. For example, both myocardial fatty acid, glucose, and ketone body oxidation rates have been shown to vary depending on the heart failure model used and the duration of heart failure. Other molecular changes may also contribute to the diverse nature of heart failure energy metabolic pathology. This includes changes to transcriptional control, post-translational modifications, and mitochondrial biogenesis, all of which occur in response to heart failure's metabolic inflexibility.

## Glucose metabolism in the failing heart

As discussed, the ischemic failing heart has a considerable energy deficit (30–40%) due to impaired mitochondrial oxidative metabolism, as well as due to the fact that there is a shortage in oxygen transport and an increased reliance on less efficient energy substrates which have a low O_2_/ATP ratio (9, 26). Importantly, other forms of heart failure, including non-ischemic failing heart, might not have the same oxygen deficit as was shown in animals (77) and human (78). This could potentially have implications on substrates metabolism, but it is out of the scope of this review. While there is a general consensus that the heart switches from fatty acid oxidation to glucose metabolism to produce energy in heart failure, we believe it is more plausible to state that the failing heart switches from mitochondrial oxidative phosphorylation to glycolysis as a main source of energy.

### Glycolysis

**Figure 2:**
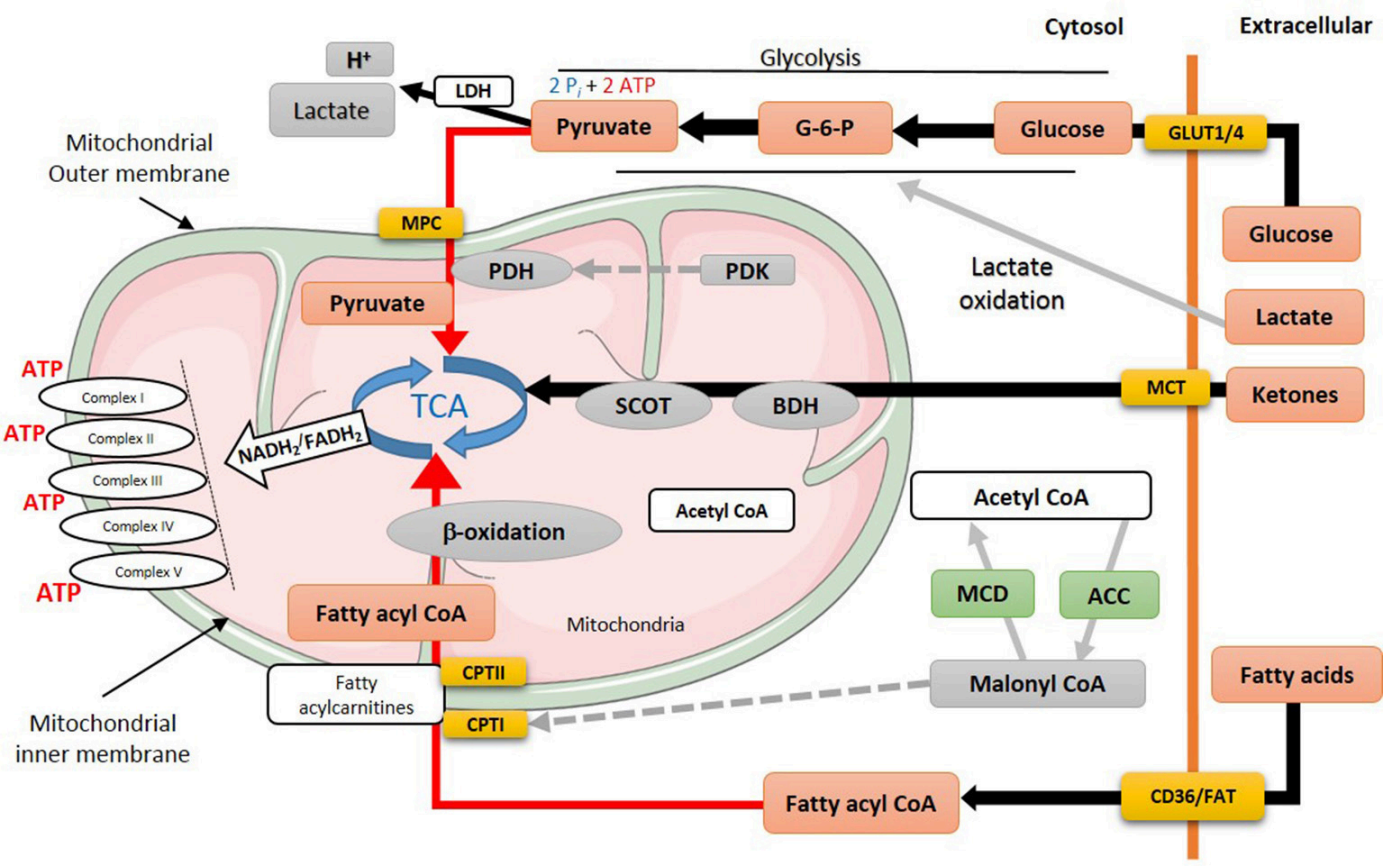
Energy metabolism in heart failure. During heart failure, overall mitochondrial oxidative metabolism and electron transport chain activity is compromised. Increased flux is indicated by black lines, while red lines indicate impaired utilization of different substrates. MPC, mitochondrial pyruvate career; PDH, pyruvate dehydrogenase; PDK, pyruvate dehydrogenase kinase; TCA, tricarboxylic acid cycle; SCOT, succinyl-CoA-3-oxaloacid CoA transferase; BDH, β-hydroxybutyrate dehydrogenase; CPT, carnitine palmitoyltransferase; MCD, malonyl CoA dehydrogenase; ACC, acetyl CoA carboxylase; MCT, monocarboxylate transporter; CD36, cluster of differentiation; FAT, fatty acid translocase; GLUT, glucose transporter type (1 or 4).

Glycolysis is anaerobic metabolic process which its rates increase in the failing heart (18) (Figure 2). Direct measurements of myocardial glycolytic rates showed a marked increase in both abdominal aorta constriction (ACC)- and transverse aortic-constriction (TAC)-induced heart failure animal models (21, 79–81). In patients with heart failure with reduced ejection fraction (HFrEF), there is a marked increase in glucose uptake and glycolysis that is associated with an increased lactate and pyruvate accumulation (82). This increase in glycolysis and the accumulation of glycolysis by-products, namely lactate, and proton, is also seen in heart failure in Dahl salt-sensitive rats fed a high salt diet (83), and in patients with heart failure (82). While glycolysis provides an anaerobic source of ATP, the accumulation of glycolysis-derived protons in the cytosol can contribute to acidosis which can reduce cardiac contractility, as it desensitizes contractile proteins to Ca^2+^ and inhibits the slow Ca^2+^ current (27, 84). It also leads to Na^+^ and Ca^2+^ overload as cardiomyocytes attempt to extrude excess proton to the extracellular space. It can also lead to an ionic imbalance which can aggravate the energy deficit seen in heart failure, as the already limited cardiac ATP supply is shifted toward re-establishing ionic homeostasis.

An important point to consider when measuring myocardial glycolytic rates is the animal model used. Employing murine hearts to address myocardial energy metabolism has increased dramatically due to the flexibility mice provide for knockdown or overexpression of target genes. The murine model, however, may not be ideal in cases where myocardial glycolytic measurements are required, especially if glycolytic rates are differentially affected due to a pathological disease. For example, heart failure-induced up-regulation in glycolysis rate is not detectable if the mouse heart is used as the model to study heart failure (13, 14, 83). This is probably due to the fact that the mouse heart is highly glycolytic compared to other animals models (85) and this high glycolysis is already saturating its contribution to overall ATP production (~20%). Therefore, differences in energy substrates utilization between different animal models need to be considered (86, 87). Nevertheless, other studies have also reported a marked increase in glycolysis rate in other animal models of heart failure (80, 88–90).

One of the characteristic changes in heart failure is the development of insulin resistance which has been shown *in vivo* (13, 14, 91–95) and in human (96–98). Insulin regulates glucose uptake by enhancing GLUT4 translocation (99, 100) and increases glycolysis (101–103). In insulin resistance in heart failure, the heart switches to GLUT1 to take up glucose. Despite this impaired insulin signaling, glycolysis is increased in the failing heart.

### Glucose oxidation

We and others have reported that impairment of glucose oxidation is a metabolic marker that precedes the development of cardiac dysfunction in different animal models of heart failure (14, 94). Although glycolysis rates increase in heart failure, this does not necessarily translate into an increase in glucose oxidation since glycolysis and glucose oxidation are differentially regulated in the heart (104). The majority of studies directly examining the failing heart's glucose oxidation rates in humans and animals show a marked decrease in glucose oxidation in the failing heart, and a reduced contribution of glucose oxidation to overall ATP production (13, 14, 91, 92, 94, 96, 98, 105). A study by Diakos et al. (82) also demonstrated that the increase in cardiac glycolysis seen in severe heart failure patients was not accompanied by an increase in lactate and pyruvate accumulation, suggesting that the increase in glycolysis is not matched by an increase in glucose oxidation. In support of this, Paolisso et al. (96) reported an abrogated rate of glucose oxidation in patients with congestive heart failure. Furthermore, impairment of pyruvate oxidation in transgenic mice is associated with the development of left ventricular hypertrophy (89), emphasizing the relationship between maintained glucose oxidation and normal cardiac function. In support of this, Kato et al. (106) showed that in Dahl salt sensitive rats with heart failure (which have high cardiac glucose uptake and glycolysis), stimulating PDH with dichloroacetate improved heart function and decreased lactate production (presumably due to an increase in glucose oxidation). Combined, these studies suggest an important role of cardiac metabolic inflexibility, which occurs in heart failure, with regards to glucose oxidation in mediating heart failure severity.

While the majority of studies suggest a decrease in glucose oxidation in the failing heart, not all studies are consistent with this finding. The extent of reduction in cardiac glucose oxidation in heart failure varies according to the severity of heart failure, as well as the experimental model of heart failure used and the availability of other energy substrates. In a rat model of transverse aortic constriction (TAC), for instance, Doenst et al. (107) showed that glucose oxidation rates remained unchanged in a rat model of compensated heart failure (due to mild TAC) glucose oxidation was only reduced after systolic dysfunction occurred. Whether the slow development of diastolic dysfunction over a relatively long period of time in animal models has an impact on energy metabolism changes needs further investigation. In support of this, Zhang et al. (14) also reported that glucose oxidation rate was only decreased as an early sign of cardiac dysfunction in another mild heart failure model induced by AAC. As the severity of heart failure increases, this will have a direct impact on glucose oxidation rate. This was shown in a number of studies where a mouse model of pressure-overload-heart failure with severe cardiac dysfunction showed a marked impaired glucose oxidation rates (93–95). However, Osorio et al. (108) reported that glucose oxidation rate was increased in a canine cardiac pacing model of heart failure which, however, contrasts the decrease in glucose oxidation seen in pacing-induced heart failure seen in pigs (88). Unfortunately, cardiac glycolytic rates were not measured in the study by Osorio et al. (108). In a TAC model of murine heart failure, an increase in the proportion of glucose oxidized by the hearts was also seen (109). The reason for these discrepant findings is unclear. However, it should be acknowledged that high pyruvate supply from increased glycolysis has the potential to augment glucose oxidation (110). Indeed, the study by Kolwicz et al. (109), which suggests that glucose oxidation was increased in TAC hearts from mice, also observed a marked increase in lactate and alanine production, indicative of increased glycolysis rates. Furthermore, while pacing-induced heart failure in dogs showed an increase in glucose oxidation (111), cardiac function was markedly low in these dogs [left ventricular work (LVW) was 148 ± 28 Hg^*^mm while normal LVW is 800–900 Hg^*^mm]. In severe heart failure, it is possible that mitochondrial calcium control is compromised, potentially leading to mitochondrial calcium accumulation, and activation of the PDH complex due to calcium activation of PDH phosphatase (112).

The reduction in glucose oxidation that occurs in the failing heart is due, in part, to an overall deterioration in mitochondrial oxidative capacity, as well as due to an impaired activity of PDH, the rate-limiting enzyme for glucose oxidation (13, 14, 92, 98). There is also some evidence that the machinery of glucose oxidation could be impaired in the failing heart (104). Gupte et al. (113) demonstrated that the mRNA expression of PDH, MCT1, and pyruvate/alanine aminotransferase, were decreased in heart failure, suggesting impairment in pyruvate metabolism. In line, Dodd et al. (89) demonstrated, using hyperpolarised ^13^C-magnetic resonance spectroscopy, that PDH complex activity is impaired in a rat model of myocardial infarction-induced heart failure. Of importance, is that PDH complex impairment was progressive and proportional to the degree of cardiac dysfunction.

As it was discussed earlier (see Glycolysis section), it is generally accepted that insulin-induced stimulation of glucose oxidation is markedly attenuated in obesity and diabetes (114, 115), contributing to myocardial metabolic inflexibility (116). Impairment in insulin signaling and development of insulin resistant myocardium precedes cardiac dysfunction in heart failure and it is a major determine of its progression (14, 94, 117). Rutter et al. (118) showed that increased insulin resistance, which is also associated with obesity, was accompanied with worsening cardiac remodeling. In consistence, Peterson et al. (119) also, in consistence, demonstrated that insulin resistance in obese women was associated with deterioration in cardiac efficiency. Taken together, it seems plausible to suggest that insulin resistance is, at least in part, responsible for the reduction in glucose oxidation in heart failure. In a rat model of streptozotocin-induced diabetic cardiomyopathy, insulin resistance is associated with a marked decrease in PDH flux and diastolic function (120). Of interest, is that dichloroacetate (DCA), a PDK inhibitor that enhances PDH activity, treatment reversed insulin resistance, increase PDH flux and improving cardiac function (120). This further emphasizes the potential therapeutic applications of improving glucose oxidation to mitigate cardiac dysfunction in heart failure. In the same context, cardiac-specific PDHA1(^−/−^) knockout mice showed impaired insulin signaling which was associated with diastolic dysfunction (121). Consistent with this, Sankaralingam et al. (115) demonstrated that cardiac dysfunction and insulin resistance were both worsened by high fat diet-induced obesity in abdominal aortic constriction-induced heart failure mice model. Collectively, this further emphasizes the crucial role of insulin resistance and high circulating FFAs in the pathogenesis of heart failure.

Another mechanism by which glucose oxidation could be attenuated in heart failure is through heart failure-induced mitochondrial hyperacetylation. Hyperacetylation occurs in the failing heart (122) and hyperacetylation of PDH has previously been shown to have an inhibitory effect on its activity (123, 124). Thus, heart-failure induced hyperacetylation could inhibit PDH activity and decrease glucose oxidation rates. It has also been reported that hyperacetylation can increase the activity of fatty acid oxidation enzymes, such as LCAD and β-HAD (125). As such, enhancing the activity of these ß-oxidation enzymes could negatively feedback to inhibit glucose oxidation through the Randle cycle (38, 126).

As discussed, the failing heart has a considerable energy deficit (30–40%) due to impaired mitochondrial oxidative metabolism, as well as due to the fact that there is a shortage in oxygen transport and an increased reliance on less efficient energy substrates which have a low O_2_/ATP ratio (9, 26). While there is a general consensus that the heart switches from fatty acid oxidation to glucose metabolism to produce energy in heart failure, we believe it is more plausible to state that the failing heart switches from mitochondrial oxidative phosphorylation to glycolysis as a main source of energy.

## Fatty acid oxidation in the failing heart

While it is generally agreed that the failing heart has reduced cardiac energetics, mitochondrial TCA cycle activity and overall oxidative metabolism (9, 70, 71), it is less clear whether myocardial fatty acid oxidation rates are also decreased. It is generally assumed that cardiac fatty acid oxidation is decreased in heart failure (10, 127–129), which is supported by decreased transcription of a number of enzymes involved in fatty acid oxidation (10, 128–132). However, direct measurements of fatty acid oxidation rates in both human and experimental models of heart failure do not always support this assumption.

Heart failure can be associated with an increase in circulating FFA levels due to high lipolysis rates (96, 133, 134). Of importance, is that increased level of circulating FFAs in failing heart is an important determinant of fatty acid oxidation rates in the heart. For example, decompensated heart failure patients show an increase in circulating FFAs levels, which is accompanied by enhanced myocardial fatty acid uptake and fatty acid oxidation (135, 136). In support of this, Taylor et al. (137) also demonstrated that fatty acid uptake rates are up-regulated in patients with severe heart failure (ejection fraction ~24%), using a positron emission tomography (PET) imaging technique. In contrast, Dávila-Román et al. (73), who also using (PET) imaging, showed a decrease in circulating FFAs level in patients with non-ischaemic heart failure. Neglia et al. (75) also showed a decreased fatty acid oxidation in patients with idiopathic dilated cardiomyopathy.

Animal studies also show differing results as to what happens to fatty acid oxidation in the failing heart. Studies in mice in which heart failure was produced secondary to pressure overload or a myocardial infarction have shown that cardiac fatty acid oxidation rates are unchanged (94, 95, 110). Mori et al. (91) also showed that fatty acid oxidation rate was not changed in Ang II-induced heart failure. However, it should be recognized that these maintained rates were seen despite a decrease in cardiac function, suggesting that fatty acid oxidation per unit work may actually increase in heart failure. In addition, these studies showed that the contribution of fatty acid oxidation to total ATP production increased, due primarily to a decreased contribution of glucose oxidation to ATP production. Others have also shown that with compensatory heart failure, fatty acid oxidation enzymes are preserved (21, 79). In contrast, Byrne et al. (94) and Sung et al. (93) have shown that cardiac fatty acid oxidation rates are impaired in TAC-induced severe heart failure in mice. In rats subjected to TAC, Doenst et al. (107) also demonstrated that fatty acid oxidation rates decreased in parallel with an overall decrease in mitochondrial oxidative phosphorylation (107). Moreover, a reduction in fatty acid oxidation rates was also seen in canine models of severe heart failure (138, 139).

The issue of what happens to fatty acid oxidation in the failing heart becomes more complex in the presence of obesity and/or diabetes is present. Even in the absence of heart failure, fatty acid oxidation rates are elevated under these conditions (119, 140–142). These high cardiac fatty acid oxidation rates persist if evidence of heart failure is seen in obesity and diabetes (115, 141, 143). Furthermore a strong link between reduced cardiac efficiency and excessive reliance on fatty acid oxidation has been shown in *ob/ob* mice (141) and obese humans (119).

The reasons for the confusion as to what is happening to fatty acid oxidation in heart failure, may be related to alterations in the control of fatty acid oxidation at multiple levels, including changes in fatty acid supply to the heart, alterations in allosteric control of fatty acid oxidation, alterations in transcriptional control of fatty acid oxidation, and alterations in post-translational control of fatty acid oxidation. Increased fatty acid supply to the heart will increase fatty acid oxidation, as will the presence of insulin resistance (12). Indeed, Tuunanen et al. (144) showed that while cardiac fatty acid oxidation rates were decreased in patients with idiopathic dilated cardiomyopathy, as cardiac function deteriorated insulin resistance occurred with a subsequent increase in fatty acid oxidation rates. As discussed, in heart failure a marked cardiac insulin resistance occurs (13, 14, 92). This includes a decreased ability of insulin to inhibit fatty acid oxidation (14, 92). In the presence of obesity and/or diabetes, this cardiac insulin resistance is even more dramatic (115). As result, an increased cardiac insulin resistance in heart failure may contribute to maintaining fatty acid oxidation rates. Heart failure is often associated with impairment in insulin signaling which could have a marked impact on energy metabolism in the heart. Insulin has an inhibitory effect on fatty acid oxidation through enhancing the activity of acetyl CoA carboxylase which increases the tissue level of malonyl CoA thereby decreasing mitochondrial fatty acid uptake. Insulin-induced inhibition of fatty acid oxidation is impaired in the failing heart leading to an increase in fatty acid contribution to the total ATP production, despite being inefficient substrate during heart failure. Furthermore, inactivation of the carnitine shuttle system increases cytosolic fatty acyl CoA levels (or long chain acyl CoA) and, in addition to the accumulation of TAG and diacylglycerol (DAG), can have a negative impact on insulin signaling (14, 145, 146). For instance, excess lipid metabolites can phosphorylate serine residues on IRS-1 by activating IKK-NF-κB, JNK-AP-1, and the PKC pathway all of which can reduce glucose uptake by decreasing Akt and PI3K activity (14, 147).

Changes at the transcriptional level of genes involved in fatty acid oxidation are often cited as a key reason why cardiac fatty acid oxidation rates may be decreased in heart failure (148, 149). A down regulated gene expression of fatty acid oxidative enzyme (LCAD, MCAD) has been observed in heart failure patients, as well as during the progression of heart failure in animal models (130). Three distinct isoforms of peroxisome proliferator activated receptor [PPARα (cardiac abundant), PPARβ/δ, and PPARγ] are responsible for the transcriptional changes of fatty acid metabolic genes (150, 151). PPAR and the retinoid X receptor (RXR) complex are transferred to the nucleus to bind with a specific PPAR response element (PPRE), which is located in the target gene's promoter. An inducible PPARγ coactivator-1α (PGC-1α) is also correlated with the transcriptional activity of PPAR superfamily (149, 152). Fatty acids are the endogenous ligand of the PPARα and may activate the PPARα/PGC1α pathway for the transcriptional regulations. PPARα/PGC1α transcriptional activity has also been shown to regulate pyruvate dehydrogenease kinase 4 (PDK4), which can reduce glucose oxidation by inactivation of PDH activity (153, 154), but not glycolysis. Again in heart failure patients, cardiac PPARa expression was shown to down regulated (113, 155). The expression of PGC-1α, important for mitochondrial biogenesis, is also down regulated in heart failure [(20, 21, 48, 156)]. In pressure-overload-induced heart failure, abnormal mitochondrial morphology and reduced mitochondrial density is seen, which is associated with altered electron transport chain proteins expression (20). Patients with heart failure also show a reduction in mitochondrial DNA contents which was accompanied by down regulation of PGC-1α-associated proteins (157). Furthermore, based on DNA microarray analysis, it has been shown that a subset of downstream gene targets of PGC-1a are also down-regulated in the failing heart, which is correlated with the reduced left ventricular ejection fraction (158). However, it is still not clear whether this attenuation in the role of PGC-1α in heart failure is enough to manipulate mitochondrial biogenesis. However, a reduction in the number of mitochondria could contribute to the changes in fatty acid oxidation observed in heart failure.

Post-translational modifications may also alter fatty acid oxidation in the failing heart This includes mitochondrial lysine acetylation, in which an acetyl group is transferred to a lysine residues or mitochondrial proteins. Acetylation can be mediated through histone and non-histone acetyl-transferase (159). Furthermore, mitochondrial acetyltransferase, namely GCN5L, has also been shown to promote acetylation (160). On the reverse reaction, sirtuins (SIRTs) act as deacetylases to reverse the effect of acetylation (161, 162). Acetylation controls the activity of number of metabolic enzymes (163). Hyperacetylation of LCAD and ßHAD results in an increase in fatty acid oxidation rates (125). In obese mice with heart failure, an elevated GCN5L expression in abdominal aortic constriction-induced heart failure is associated with increased in LCAD acetylation and an increase in fatty acid oxidation (115). Furthermore, switching to a low fat diet in obese mice showed the opposite effect on the post-translational modification associated with reduced fatty acid oxidation (115). Moreover, we also showed glucose oxidation could be inhibited through hyperacetylation in heart failure (92). Taken together, post-translational modifications may be another factor to be considered which might to explain the metabolic inflexibility during heart failure.

## Insulin resistance and heart failure

Glucose and fatty acid metabolism are tightly controlled by insulin signaling in the heart. Evidence from clinical studies have shown a strong association between insulin resistance and cardiac dysfunction (118, 164). Moreover, patients with insulin resistance have high rates of lipolysis in adipose tissue with increases in TAG hydrolysis (164–166). Of importance, insulin resistance-induced shifts in favor of fatty acid oxidation and is associated with attenuation of glucose uptake by the heart [see review by (116)]. This change in metabolic preference was also observed in an experimental setting (13, 14, 91, 167) and clinical studies of heart failure (82, 88). Of interest, insulin resistance precedes any changes in cardiac energy metabolism in mice subjected to abdominal aortic constriction (14). Impairment in insulin signaling primarily has a direct inhibitory effect on fatty acid oxidation by increasing the malonyl CoA levels and secondarily inhibiting glucose oxidation through a negative feedback effect of the Randle cycle (38). Moreover, increasing fatty acid oxidation rates could also indirectly cause attenuation in glucose oxidation by triggering the activity of PDK and limiting the activity of the PDH complex. In a model of angiotensin II-induced heart failure with preserved ejection fraction (HFpEF), glucose oxidation rates were reduced by 45% as PDK activity was enhanced and PDH complex activity was attenuated (91). These results are further supported as PDK deletion improved the HFpEF-induced reduction in glucose oxidation (92).

## Ketone oxidation in the failing heart

### Circulating ketone body concentrations in heart failure

A major determinant of ketone oxidation rates in the heart are the levels of circulating ketones. Earlier studies have suggested that blood ketone levels are elevated in congestive heart failure (with reduced ejection fraction) patients proportional to the severity of cardiac dysfunction (168, 169). These results were recently challenged by Melenovsky et al. (170) who found that the plasma level of ßOHB in heart failure patients were similar to healthy subjects. However, in support of the earlier observations by Lommi et al. (168) and Lommi et al. (169), recent metabolomics studies have found increased blood ketone levels in HFrEF patients (171) and HFpEF patients (172). It is interesting to note however that Zordoky and colleagues also found that HFpEF patients had significantly higher blood ketone levels than HFrEF patients while HFrEF patients had lower ketone levels than healthy controls (172). The discrepancy in reported levels of circulating ketones in heart failure patients may be due to multiple reasons including differences in severity, duration and type of heart failure. This is especially the case in severe heart failure where insulin resistance-induced increases in hepatic ketogenesis could be inevitably contributing to increases in circulating ketones (173). In addition, it is important to note that cardiac ketone levels are dynamic. In general, the circulating levels and cellular uptake of ketones is proportional to its contribution to ATP production (174). In that regard, it is difficult to pin point cardiac ketone levels without concurrently considering pathological circulating serum levels, uptake, oxidative rates and secretion rates of ketone (175). For example, it is still not clear whether HFrEF patients, with lower blood ketone levels than HFpEF patients (172), have a greater reliance on ketone bodies and increased myocardial ketone body oxidation. Alternatively, it is possible that HFpEF patients with high circulating ketone body levels could be associated with increased muscle uptake and oxidation of ketone bodies. Therefore, changes in circulating ketone body levels are temporal, indefinite, and direct measurements of flux through myocardial ketone body oxidation rates are required.

### Ketone body oxidation in heart failure

Arterio-venous measurements for ßOHB in HFrEF patients, a surrogate for myocardial ketone body utilization, reported no differences compared to healthy controls (176), and a slight increase, however not significant, in HFrEF patients (98). However, recent studies have suggested that myocardial ketone body oxidation is increased in heart failure. In a mouse model of compensated and decompensated pressure overload cardiac hypertrophy, proteomics data demonstrated that a key enzyme involved in ketone body oxidation, namely ß-hydroxybutyrate dehydrogenase (BDH1), was up-regulated 2 to 3-fold (15). Moreover, the myocardial metabolite profile of mice with heart failure was comparable to mice fed a 4-week ketogenic diet. In parallel, Bedi et al. (16) also observed an increased ratio of serum to myocardial ketone bodies with up-regulated expression of BDH1, BDH2, and succinyl-CoA:3-ketoacid CoA transferase (SCOT) in human heart failure patients. We have also seen an increase in myocardial ketone body oxidation rates in the *ex vivo* isolated failing murine heart (unpublished data) (17). Taken together, these studies suggest that the failing heart has an increased reliance on ketone body oxidation. Nevertheless, Nagao et al. (177) found that βOHB levels were elevated in mice with ascending aortic banding-induced heart failure. *In vitro*, subjecting cardiomyocytes to oxidative stress also resulted in elevated levels of βOHB, increased levels of anti-oxidative factors, and concurrent down-regulation of the rate limiting enzyme in ketone body oxidation, SCOT (177). These findings would suggest that ketone body oxidation decreases during heart failure to maintain elevated levels of βOHB as a compensatory response to protect the heart against oxidative stress. The reason for the contradictory results could be due to the severity and duration of heart failure in these studies (15, 177). Since heart failure is a chronic condition, it seems plausible to suggest that in the early stage of heart failure, βOHB may have an antioxidant role and only become an adaptive fuel source in the end-stage heart failure (177). It is worth mentioning that βOHB has previously been shown to be an HDAC inhibitor and protects against oxidative stress (178). However, with this uncertainty comes the question of whether ketone body oxidation in heart failure is adaptive or maladaptive (23, 173)?

To address whether ketones are adaptive or maladaptive in failing hearts, several recent studies have investigated this. Schugar et al. (179) reported that mice with a cardiac-specific knockout of *Oxct1* (or SCOT, the rate limiting enzyme in ketone body oxidation) subjected to TAC-induced heart failure was associated with increased rates of anaplerosis, mitochondrial ultrastructure abnormality and accelerated pathological cardiac remodeling. Similarly, overexpression of cardiac BDH1, the first enzyme in the ketone body oxidation pathway, mitigated oxidative stress and attenuated cardiac remodeling following pressure overload-induced hypertrophy (180). Together, these studies suggest that heart failure-induced increases in ketone body oxidation are adaptive for a failing heart. However, there are still several aspects to consider in light of the preliminary suggestion that ketone body oxidation is adaptive in the setting of heart failure. One factor to consider is the change in cardiac ketone levels, a dynamic concentration that would decrease if heart failure is characterized by elevated myocardial ketone body oxidation rates. Keeping this in mind, the up-regulation of cardiokine/myokine follistatin-like protein 1 (FSTL1), having been shown to be cardioprotective in heart failure, is accompanied by a reduction in cardiac ketone body uptake in a canine model of tachypacing-induced heart failure (181). In such a scenario, high cardiac ketone levels would be suggested to be undesirable and accelerating myocardial ketone body oxidation would be adaptive in heart failure. Alternatively, since ßOHB has been shown to be an HDAC inhibitor (178) and recent work has demonstrated that HDAC inhibitors can enhance myofibril relaxation kinetics and improve diastolic function (182), maintaining ketone levels may indeed be beneficial in the setting of HFpEF as opposed to HFrEF. Second, another factor to consider are post-translational modifications which may be responsible for myocardial energy metabolic derangements that contribute to the progression of heart failure (122). In this case, increasing myocardial ketone body oxidation and increasing the myocardial acetyl CoA pool has been suggested to provide more substrate for lysine acetylation, ultimately contributing to the failing heart's hyperacetylated state (122). This may or may not be desirable since hyperacetylation of glucose and fatty acid oxidation enzymes (183), and its effects on enzyme activity, require further investigation in the setting of heart failure.

### Ketones' effects on glucose and fatty acid oxidation

Ketones have the potential to suppress glucose oxidation and *vice versa* (37, 69, 184) as they both compete for available oxygen and as a source of TCA cycle acetyl CoA. Williamson and Krebs (184) observed that in the presence of insulin, acetoacetate decreased glucose oxidation by half in the perfused rat heart. This may be explained by the ability of ketones to increase the mitochondrial acetyl-CoA to CoA ratio and consequently inhibit the activity and flux through PDH, the rate limiting enzyme of glucose oxidation (185–187). Of importance is whether ketones add a new dimension of complexity to the Randle cycle (38). Furthermore, the influence of ketone levels and its inhibitory effect on glucose oxidation also needs to be characterized to understand whether it is beneficial to enhance either of these pathways. Recently, ketone bodies were found to decrease myocardial glucose uptake and increase myocardial blood flow in a PET study in healthy humans (188). In connection with this displaced glucose uptake, leucine metabolism into ketone bodies has also been shown to inhibit GLUT4 translocation in cardiomyocytes due to an increase in lysine acetylation, ultimately hampering cardiac glucose uptake (189). Since heart failure is characterized by an increase in acetylation (122), the failing heart's hyperacetylated state could be potentiating leucine to ketone-mediated GLUT 4 inhibition and partially conferring insulin resistance. However, this is in contrast to a study that showed that administration of ßOHB and acetoacetate were able to recapitulate insulin-induced improvements in an isolated perfused rat heart's *ex vivo* cardiac efficiency (190). Since the failing heart is insulin resistant, ketones may be a viable substrate to improve cardiac efficiency in the failing heart (190). However, more studies measuring ketone body oxidation flux in the presence and absence of insulin are required.

It has also been reported that ketones can inhibit myocardial fatty acid oxidation (191, 192). For example, intravenous infusion of ßOHB suppressed myocardial fatty acid oxidation independent of changes in malonyl-CoA levels or the ratio of acetyl-CoA to CoA in pigs (193). Since fatty acid oxidation rates in heart failure remains controversial, it is unclear whether the inhibitory effects of ketones on myocardial fatty acid oxidation are beneficial or detrimental for the failing heart. This, however, further underlines the crucial role of ketone bodies in myocardial energy metabolism and implicates ketones as an important role-player that is currently neglected from the Randle cycle.

## Therapeutic approaches to address the failing heart's metabolic profile

### Stimulating glucose oxidation

Targeting glucose metabolism has been shown to be an effective approach to mitigate cardiac remodeling and improve heart function. Ikegami et al. (117) and Liao et al. (194) both reported that enhancing glucose uptake by GLUT1 and GLUT4 improved cardiac function and attenuated pressure-overload-induced hypertrophy in mice. Dichloroacetate (DCA) is a direct PDK inhibitor which increases glucose oxidation via enhancing PDH complex activity in the setting of heart failure. In the isolated working rat heart DCA enhances post-ischemic cardiac function and efficiency which is associated with improved coupling between glycolysis and glucose oxidation (195). DCA-induced improvement in coupling between glycolysis and glucose oxidation was also later demonstrated in suprarenal abdominal aortic constriction in rat (196). Similarly, Kato et al. (106) demonstrated that DCA administration to Dahl-salt sensitive rats increased cardiac energy reserve, reduced oxidative stress and slowed the transition from compensated heart failure to failing heart. In line with experimental studies, small clinical studies, although few, have shown promising improvement in cardiac contractility with DCA treatment in patients with coronary artery disease (197) and heart failure (198). Clinical data were not all consistent as DCA infusion in patients with congestive heart failure did not show significant beneficial effects (199).

### Inhibiting fatty acid oxidation

**Figure 3:**
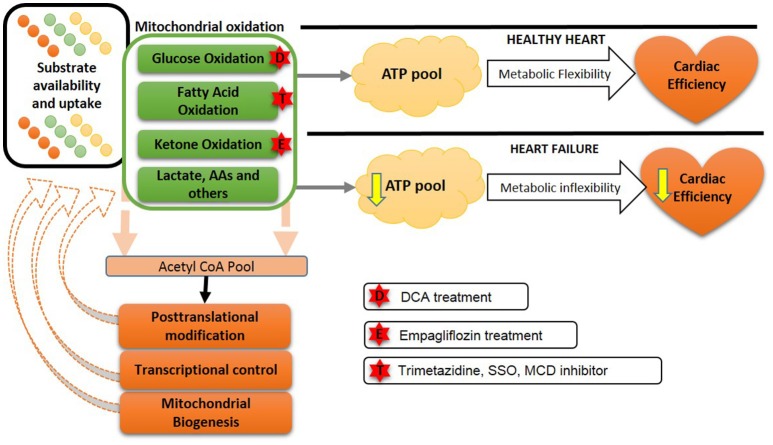
Future approaches to overcome the metabolic balance or inflexibility: In the healthy heart, a variety of energy substrates produce ATP to maintain metabolic flexibility and cardiac efficiency. However, in heart failure a reduced ATP production occurs due to a decreased metabolic inflexibility and a less efficient heart. Transcriptional changes and altered mitochondrial biogenesis also contribute to this metabolic inflexibility in heart failure. Possible approaches to improve metabolic flexibility are shown by stars. DCA, dichloroacetate; SSO, sulfo-N-succinimidyl-oleate; MCD, malonyl CoA dehydrogenase; AA, amino acids.

There are number of pharmacological approaches which are shown to successfully reduce fatty acid oxidation. Two molecules, namely etomoxir and perhexiline, is shown to inhibit CPT1 (Figure 3) and limit fatty acid oxidation with parallel increase in glucose oxidation in mouse and rat models of heart failure (200, 201). In humans, etomoxir showed improvement in ejection fraction and cardiac output (202, 203). Perhexiline also improves cardiac function and symptoms of heart failure (204) However, clinical trials to validate the preliminary encouraging finding with these two molecules were terminated due to the hepatotoxicity (204).

Furthermore, sulfo-N-succinimidyl-oleate (SSO) is an inhibitor of CD36 and has been shown to decrease fatty acid oxidation followed by an indirect increase in glucose oxidation (205). While these approaches showed beneficial effects in experimental studies involving heart failure, clinical studies with these agents have yet to be performed.

Another approach to inhibiting fatty acid oxidation is to inhibit the last enzyme of fatty acid ß-oxidation, 3-keotacyl CoA thiolase, with trimetazidine (206). Clinically trimetazidine has shown beneficial effects by increasing cardiac efficiency, where there is a shift in myocardial substrate utilization from fatty acid oxidation to glucose oxidation. It has been using as an antianginal agent in more than 100 countries (207). A combination of chronic trimetazidine treatment along with the other conventional therapy in heart failure patients improves cardiac function in humans (208). Treatment with trimetazidine in heart failure patients with idiopathic dilated cardiomyopathy shows a decrease in myocardial fatty acid oxidation rates, as well as improved left ventricular function and insulin sensitivity (208). A meta-analysis of clinical trials with trimetazidine in heart failure, showed a beneficial effect of trimetazidine on left ventricular systolic function, clinical symptoms for patients with chronic heart failure and importantly may result in decreasing all-cause mortality (209). We have also shown that trimetazidine prevents obesity-related reductions in cardiac function in obese mice with heart failure (210, 211).

Another potential intervention to decrease fatty acid oxidation in heart failure is with ranolazine. Ranolazine has been clinically used as anti-anginal agent since 2006 (212, 213). While considered to be an inhibitor of the late Na^+^ current, ranolazine is also a fatty acid oxidation inhibitor, which is capable of activating PDH a rate limiting enzyme for glucose oxidation (214). However, ranolazines efficacy in treating heart failure has not been extensively studied.

### Enhancing ketone body oxidation

In diabetic cardiomyopathy, ketones have been popularized as a thrifty fuel substrate for the heart (215, 216). The role of myocardial ketone metabolism has attracted huge attention since empagliflozin, a sodium glucose co-transporter-2 inhibitor used to treat type 2 diabetes, has shown cardioprotective effects in type 2 diabetic patients. This cardioprotection was associated with increased plasma ketone levels which led to the proposed cardioprotective role of increased ketone body oxidation in the diabetic failing heart (215–217). Furthermore, a recent study found that non-diabetic mice with experimental TAC-induced heart failure was protected against heart failure-induced decreases in *in vivo* and *ex vivo* function following a 2-week treatment with empagliflozin (218). However, despite the promising and exciting findings, it is still not clear whether empagliflozin increases ketone oxidation in the heart, or whether empagliflozin-mediated cardioprotection is through a ketone-independent mechanism (219). Therefore, future studies are required to elucidate whether empagliflozin's cardiovascular benefits are mediated by changes in myocardial ketone oxidation.

## Future directions

There is a growing recognition and understanding of the importance of metabolic flexibility of the heart and how metabolic inflexibility in heart failure could contribute or even cause deterioration of cardiac contractility and affect the disease progression. Of importance, is that metabolic inflexibility could also be influenced by other comorbidities such as diabetes, obesity, hyperlipidemia and hypertension. Taken together, aiming to re-establish metabolic flexibility in the failing heart is shown to be an effective approach to improve cardiac function and therapeutic outcome. In addition, it is important to recognize the need for a “tailored therapy” for different categories of patients with heart failure based on the type and severity of heart failure as well as the comorbidities which co-exist.

Here, we will discuss some of the recent pharmacological interventions which could have clinical value for failing heart patients.

### Glucose oxidation

The shift toward utilizing a more oxygen-efficient substrate, namely glucose, could potentially have favorable effects in terms of the energy production and cardiac function of the ischemic failing heart which is oxygen deficient. This approach would also improve the coupling between glycolysis and glucose oxidation and produce more ATP per mole of glucose oxidized. It would also limit glycolysis-induced acidosis and its consequent inhibitory effect on cardiac contractility. As discussed earlier, DCA is shown to increase the contribution of glucose to the total ATP production through increasing the glucose oxidation rate with a secondary reduction in fatty acid oxidation in different experimental models and in pilot human studies. However, it is important to note that DCA has a poor pharmacokinetic profile (short half-life) and a low potency. Therefore, future investigations using DCA treatment should potentially consider continuous infusion to administer DCA to maintain effective concentration.

### Fatty acid oxidation

In the same context of utilizing oxygen efficient energy substrate in a failing heart which is under oxygen deficit, enhancing cardiac function could be achieved by reducing the reliance of the heart on fatty acid oxidation. While a considerable number of approaches exist that can directly or indirectly inhibit fatty acid oxidation, clinical trials targeting a reduction of fatty acid uptake or enzymatic activity are either limited with the confounding factors or underpowered. Therefore, framing animal models along with potential drug targeting fatty acid oxidation to optimize its effective dose, length and other possible side effects, under different diseases states (i.e. obesity, diabetes), different age, and sex should be a prime consideration prior to design future heart failure clinical studies. Unlike etomoxir and perhexiline (CPT1 inhibitors), it is not known if SSO cause hepatotoxicity. Another possible therapeutic approach is using malonyl CoA decarboxylase (MCD) inhibitors. It has been shown that by increasing malonyl CoA levels, fatty acid oxidation is reduced with a compensatory increase in glucose oxidation (25, 220). Inhibition of MCD, using the novel compound CBM-301106, increases cardiac malonyl CoA levels and decreases fatty acid oxidation (221). However, MCD inhibitors have yet to be tested in the clinical

Furthermore, inhibition of beta-oxidation enzymes, such as 3-keotacyl CoA thiolase, also has potential in reducing fatty acids oxidation l. Based on the promising outcomes in animal and human studies (described in Therapeutic Approaches section), modulation of fatty acid oxidation using trimetazidine is a potential approach to treating heart failure. Again, large randomized clinical trials are still needed to confirm this.

Of importance, is that modulating a particular energy substrate use byu the mitochondria to enhance the overall oxidative phosphorylation could be hindered by a decrease in the number and quality of the mitochondria in the myocardium. Targeting PGC1α to enhance mitochondrial biogenesis and improve the transcriptional changes in the failing heart could potentially have a therapeutic application in heart failure. Furthermore, a combination of the potential therapeutic components (trimetazidine, CD36 inhibitors, MCD inhibitors, PDK inhibitors), targeting both fatty acid and glucose oxidation during heart failure could potentially restore metabolic inflexibility and improve cardiac function in heart failure.

### Ketone oxidation

Stimulating ketone oxidation has been proposed as a potential approach for improving cardiac function in the failing heart (180). Increasing myocardial ketone oxidation has also been indirectly implies to be beneficial in the context of diabetic cardiomyopathy through empagliflozin's “thrifty fuel hypothesis.” However, there are presently no cardiac-specific drugs that can specifically modulate myocardial ketone body metabolism. While ketogenic diets are available, the extraneous systemic effects and cardiovascular risk factors associated with these high-fat diets need to be assessed as it may not be appropriate for heart failure patients. Furthermore, in light of recent work suggesting that increased ketone body oxidation is adaptive in the setting of heart failure (179, 180), increasing myocardial ketone body oxidation may be desirable, assuming it is not doing so at the cost of displacing glucose uptake, glucose oxidation or fatty acid oxidation. This would be undesirable in the context of an already depressed glucose oxidation (see section “Glucose Metabolism in Heart Failure”). Therefore, future studies characterized by normalizing ketone body oxidation in the setting of heart failure need to be conducted with measurements of the effects on other substrates and its overall cardiac energy metabolic consequences.

### Targeting the mitochondria

Recognising the central role of the mitochondria in energy metabolism and how impaired oxidative phosphorylation influences the progression of heart failure, targeting the mitochondria is another approach to regain metabolic flexibility and to improve cardiac function. Preclinical studies using mitochondrial-targeted antioxidants, such as AP39 and elamipretide, can preserve mitochondrial integrity through a marked reduction in mitochondrial ROS generation, which is associated with an improved post-infarction cardiac function *in vivo* (222, 223). Consistent with this, chronic treatment with elamipretide mitigates cardiac dysfunction in an advance heart failure model in dog, induced by a serial intracoronary microembolizations, which is accompanied with enhanced mitochondrial respiration and ATP production (224). Very recently, a double-blind, placebo-controlled clinical trial using elamipretifde in patients with HPrEF (ejection fraction ≤ 35%) showed a good tolerability and safety profile of the employed dosing range (225), encouraging future studies to characterize long-term safety and efficacy.

### Targeting cardiac contractility

As contraction and metabolism are so inextricably linked, another approach to improve cardiac metabolism is through “rationalization of ATP usage.” This approach could involve more reliance on less ATP-consuming processes to maintain ionic homeostasis and efficient utilization of the limited ATP to provide more ATP for cardiac contraction. For example, cytoplasmic Ca^2+^ handling which is mainly governed by the sarcoplasmic reticulum (SR) ATPase (SERCA2a) activity, which requires ATP to transfer Ca^2+^ into the SR. Therefore, enhancing the Ca^2+^ sensitivity of the myofibrils, which is impaired due to glycolysis-induced acidosis, could not only improve cardiac contractility but also reduce energy cost of contractility through reducing the amount of ATP used for Ca^2+^ homeostasis. Levosimendan is a Ca^2+^ sensitizer and it is shown to improve cardiac contractility in animal (226) and human (227), through enhancing Ca^2+^ sensitivity of troponin C without affecting Ca^2+^-influx. Following small clinical trials have shown that levosimendan infusion improved left ventricle contractility in different types of heart failure including congestive (228), decompensated (229) advanced/end-stage (230–232) heart failure. Future large clinical trials are warranted to validate the promising effect of Levosimendan in failing heart patients.

## Conclusions

Due to the heart's constant high energy demand, a fine balance between energy substrate utilization is crucial in maintaining metabolic flexibility. The metabolic profile of the failing heart is not simply a shift from “fatty acids to glucose.” Rather, the failing heart can be considered to have increased rates of glycolysis, depressed glucose oxidation rates and increased ketone body oxidation rates. With regards to the controversial nature of fatty acid oxidation, while the genes involved in fatty acid oxidation are down-regulated, direct measurements of rates have presented conflicting results. Thus, future studies that consider the transcriptional regulation, post-translational modifications (acetylation), absolute metabolic rates, and mitochondrial biogenesis are all required to fully understand the way in which fatty acid oxidation is perturbed in heart failure. Finally, definitively characterizing the metabolic profile of the failing heart will help direct future pharmacological therapies that can combine approaches to harmonize and normalize the metabolic flexibility of the failing heart.

## Author contributions

QK, GU, KH, and GL designed the literature search strategies and contributed to the critical analysis and interpretation of the published data. QK, GU, and KH carried out the literature search, collected the data and wrote the manuscript which was edited by GL and approved my all authors.

### Conflict of interest statement

The authors declare that the research was conducted in the absence of any commercial or financial relationships that could be construed as a potential conflict of interest.
